# Evaluating Safety and Efficacy of Follow-up for Patients With Abdominal Pain Using Video Consultation (SAVED Study): Randomized Controlled Trial

**DOI:** 10.2196/17417

**Published:** 2020-06-15

**Authors:** Dinesh Visva Gunasekeran, Zhenghong Liu, Win Jim Tan, Joshua Koh, Chiu Peng Cheong, Lay Hong Tan, Chee Siang Lau, Gaik Kheng Phuah, Newsie Donnah A Manuel, Che Chong Chia, Gek Siang Seng, Nancy Tong, May Hang Huin, Suzette Villaluna Dulce, Susan Yap, Kishanti Ponampalam, Hao Ying, Marcus Eng Hock Ong, R Ponampalam

**Affiliations:** 1 Department of Emergency Medicine Singapore General Hospital Singapore Singapore; 2 Yong Loo Lin School of Medicine National University of Singapore Singapore Singapore; 3 Health Services Research Center Singhealth Services Singapore Singapore; 4 Health Services and Systems Research Duke–National University of Singapore Medical School Singapore Singapore

**Keywords:** digital health, teleconsultation, video consultation, telereview, abdominal pain, primary care, emergency department

## Abstract

**Background:**

The benefits of telemedicine include cost savings and decentralized care. Video consultation is one form that enables early detection of deteriorating patients and promotion of self-efficacy in patients who are well but anxious. Abdominal pain is a common symptom presented by patients in emergency departments. These patients could benefit from video consultation, as it enables remote follow-up of patients who do not require admission and facilitates early discharge of patients from overcrowded hospitals.

**Objective:**

The study aimed to evaluate the safety and efficacy of the use of digital telereview in patients presenting with undifferentiated acute abdominal pain.

**Methods:**

The SAVED study was a prospective randomized controlled trial in which follow-up using existing telephone-based telereview (control) was compared with digital telereview (intervention). Patients with undifferentiated acute abdominal pain discharged from the emergency department observation ward were studied based on intention-to-treat. The control arm received routine, provider-scheduled telereview with missed reviews actively coordinated and rescheduled by emergency department staff. The intervention arm received access to a platform for digital telereview (asynchronous and synchronous format) that enabled patient-led appointment rescheduling. Patients were followed-up for 2 weeks for outcomes of service utilization, efficacy (compliance with their disposition plan), and safety (re-presentation for the same condition).

**Results:**

A total of 70 patients participated, with patients randomly assigned to each arm (1:1 ratio). Patients were a mean age of 40.0 (SD 13.8; range 22-71) years, predominantly female (47/70, 67%), and predominantly of Chinese ethnicity (39/70, 56%). The telereview service was used by 32 patients in the control arm (32/35, 91%) and 18 patients in the intervention arm (18/35, 51%). Most patients in control (33/35, 94%; 95% CI 79.5%-99.0%) and intervention (34/35, 97%; 95% CI 83.4%-99.9%) arms were compliant with their final disposition. There was a low rate of re-presentation at 72 hours and 2 weeks for both control (72 hours: 2/35, 6%; 95% CI 1.0%-20.5%; 2 weeks: 2/35, 6%, 95% CI 1.0%-20.5%) and intervention (72 hours: 2/35, 6%; 95% CI 1.0%-20.5%; 2 weeks: 3/35, 9%, 95% CI 2.2%-24.2%) arms. There were no significant differences in safety (*P*>.99) and efficacy (*P*>.99) between the two groups.

**Conclusions:**

The application of digital telereview for the follow-up of patients with abdominal pain may be safe and effective. Future studies are needed to evaluate its cost-effectiveness and usefulness for broader clinical application.

**Trial Registration:**

ISRCTN Registry ISRCTN28468556; http://www.isrctn.com/ISRCTN28468556.

## Introduction

Existing literature on telemedicine suggests that it enables cost savings and improved health care access for patients with diverse illnesses [1,2]. These reports have fueled a rise in the adoption of telemedicine for these applications in various clinical settings [3], allowing new models for decentralized care that may help alleviate shortages in health care resources and help encourage self-management by patients where appropriate [4]. This need has been heightened with the coronavirus disease 2019 (COVID-19) pandemic which has been placing many health systems in dire straits. The application of video consultation to facilitate early discharge and subsequent remote follow-up of relatively well patients is a potential solution for increasingly oversubscribed emergency departments [5], given the detrimental effects of overcrowding on the timeliness and the quality of care [6].

Patient and provider acceptance of video consultation has grown in recent years, and over two-thirds of respondents to a survey of patient and caregiver acceptance conducted in the emergency department of Singapore General Hospital were comfortable using mobile technology to share information [7]. Prior studies in Singapore have reported benefits from telemedicine such as improved health care access in the form of remote consultation mediated by health professionals for acute illnesses such as poisoning [8] or maritime emergencies [9]; however, few international studies have reported controlled outcomes from the use of telemedicine for acute illnesses by patients directly. Furthermore, many studies had considerable limitations in terms of study design [10] and clarity in reporting which telemedicine interventions were used [11].

The use of video consultation for telereview has not been conclusively investigated in patients with acute gastrointestinal ailments [12,13]. The objective of this study was to evaluate the safety and efficacy of digital telereview for patients in the emergency department who present with undifferentiated acute abdominal pain. In this study, digital telereview was evaluated as a “pull-from-patient”, a patient-led form of service delivery, and was compared with existing telephone-based telereview evaluated as a “push-to-patient”, a provider-led form of service delivery. This was the first direct and pragmatic trial of these alternative forms of service delivery for the follow-up of patients with abdominal pain.

## Methods

### Study Design

The SAVED (Safety and Efficacy of Follow-up for Patients With Abdominal Pain Using Video Consultation) study was a prospective randomized controlled trial with a 1:1 allocation ratio. An existing telephone-based telereview service (control) was compared with digital telereview using DoctorBell—a novel, web-based telehealth platform (intervention). This study was approved by the Centralized Institutional Review Board of SingHealth Singapore General Hospital (protocol number 2017/2049) and conducted in accordance with the Declaration of Helsinki (2000).

### Study Setting

The emergency department was selected as the location for this pilot study because 24-hour medical services were available to attend to any patients whose condition deteriorated [13]. Patients from the emergency department observation ward with severe undifferentiated illnesses that did not meet clinical criteria for hospital admission were recruited. This cohort represents a segment of the population of patients in the emergency department for whom the decision (final disposition) is delayed by clinical uncertainty [14,15]; therefore, the recruitment of patients discharged from the emergency department observation ward allowed investigators to stress-test the appropriateness of disposition assigned by digital telereview as well as patient adherence to recommendations, since telephone-based telereview is routinely conducted within 48 to 72 hours of discharge for patients with undifferentiated illness as a fail-safe measure that enables early detection of patients with deteriorating clinical condition. This practice improves care continuity while enabling safe discharge to address overcrowding.

### Study Recruitment

Patients with undifferentiated acute abdominal pain who were discharged from a tertiary hospital in Singapore using detailed criteria (Abdominal Pain or Gastroenteritis Pathways in Multimedia Appendix 1) were considered for recruitment at the point of discharge by study team members after the patient had received routine treatment and a disposition plan which included counselling for self-efficacy and monitoring at home as well as education regarding clinical features that warrant a return to the emergency department (re-presentation).

From September 2017 to May 2018, consecutive patients who presented to the emergency department and who satisfied the study criteria were recruited. Inclusion criteria were the ability to read in English and the ability to operate smartphone messaging apps (such as WhatsApp). To exclude patients who may be considered vulnerable, exclusion criteria were defined as less than 21 years of age, pregnant, a prisoner, cognitively impaired, or requiring a legal representative for informed consent in Singapore. As a pilot, formal sample size calculation was not possible due to a lack of relevant data.

### Randomization and Masking

After completion of informed consent, participants were randomly assigned by a study team member who withdrew lots from a box containing equal numbers of paper indicating either control or intervention. Lots in the box were replenished after each draw by a study team member (S.Y.) who was not involved in clinical care or the implementation of the randomization. At recruitment, study team members provided participants with links to web-based surveys about symptoms to be reported by patients at initial presentation and at follow-up, following recruitment and telereview, respectively. Blinding was not possible in this study.

### Intervention

Follow-up after discharge by telereview is routinely conducted by triage nurses to facilitate early discharge from the observation ward, with follow-up review to ascertain right-siting through prompt identification of deteriorating patients as well as encouraging self-efficacy in well patients who are anxious but who do not need to re-visit the emergency department. During telereview, patients who are well are advised on self-management while patients with ominous symptomatology or deteriorating illness are advised to return to the emergency department for further evaluation. In this study, patients in the control arm received routine, provider-led telephone-based telereview after discharge. Telereview was conducted by the emergency department staff on-shift at the time of the patient’s booked appointment. Any missed telereviews were actively and manually rescheduled by staff with three attempted phone calls within 48 to 72 hours following discharge. No further attempt was made to contact patients who were not reached in the 72-hour period or those who declined telereview.

Patients in the intervention arm had access to DoctorBell, a novel telehealth platform accessible on smartphone or desktop by web browsers. This was designed using a design-thinking process based on the context and workflows of an emergency department. It allowed patient-led booking, rescheduling, or cancellation of one digital telereview appointment based on the patient’s own individual availability, restricted to 48- to 72-hour window following discharge from the emergency department. Digital telereview appointments through this platform were patient-led and were not actively rescheduled by emergency department staff if missed or cancelled. Before the digital telereview appointment (synchronous teleconsultation with video, voice, and text messaging), patients received an in-app form that allowed them to report important history or symptoms beforehand (asynchronous). This was sent to the emergency department staff on-duty before video consultation, providing them with the opportunity to clarify any uncertainties with the on-duty attending physician before synchronous consultation. Upon starting work, staff could view digital telereview bookings scheduled during their shift and received automated real-time push notifications of any changes made to appointments.

### Study Outcomes and Statistical Analysis

Patients in both groups were followed up for 2 weeks to examine the study outcomes of service utilization (telereview appointments used), efficacy (patient compliance with their final disposition plan), and safety (re-presentation to the emergency department for the same medical problem within 72 hours or within 2 weeks). Initially, service utilization and safety were the only outcome measures planned for investigation; however, study team members observed that not all patients complied with instructions given during telereview to return to the emergency department. Therefore, efficacy was later added to the analysis as an outcome measure using existing data. No change to study procedures was required. For patients who did not receive telereview, the final disposition plan was that given at the point of discharge from the emergency department (ie, self-management and monitoring at home). For patients who received telereview, the final disposition plan was that given during telereview (ie, whether to continue self-management or return to the emergency department).

The study population was analyzed based on intention-to-treat. All hypotheses were two-sided with a *P*<.05 considered statistically significant. Descriptive statistical analysis was conducted using SPSS software (version 20.0; IBM Corp). Associations between categorical variables were analyzed using Fisher exact test. Where expected counts within all categories were greater than 5, chi-square test was used instead. Associations between continuous variables were analyzed using the two-tailed two-sample *t* test.

## Results

**Figure 1:**
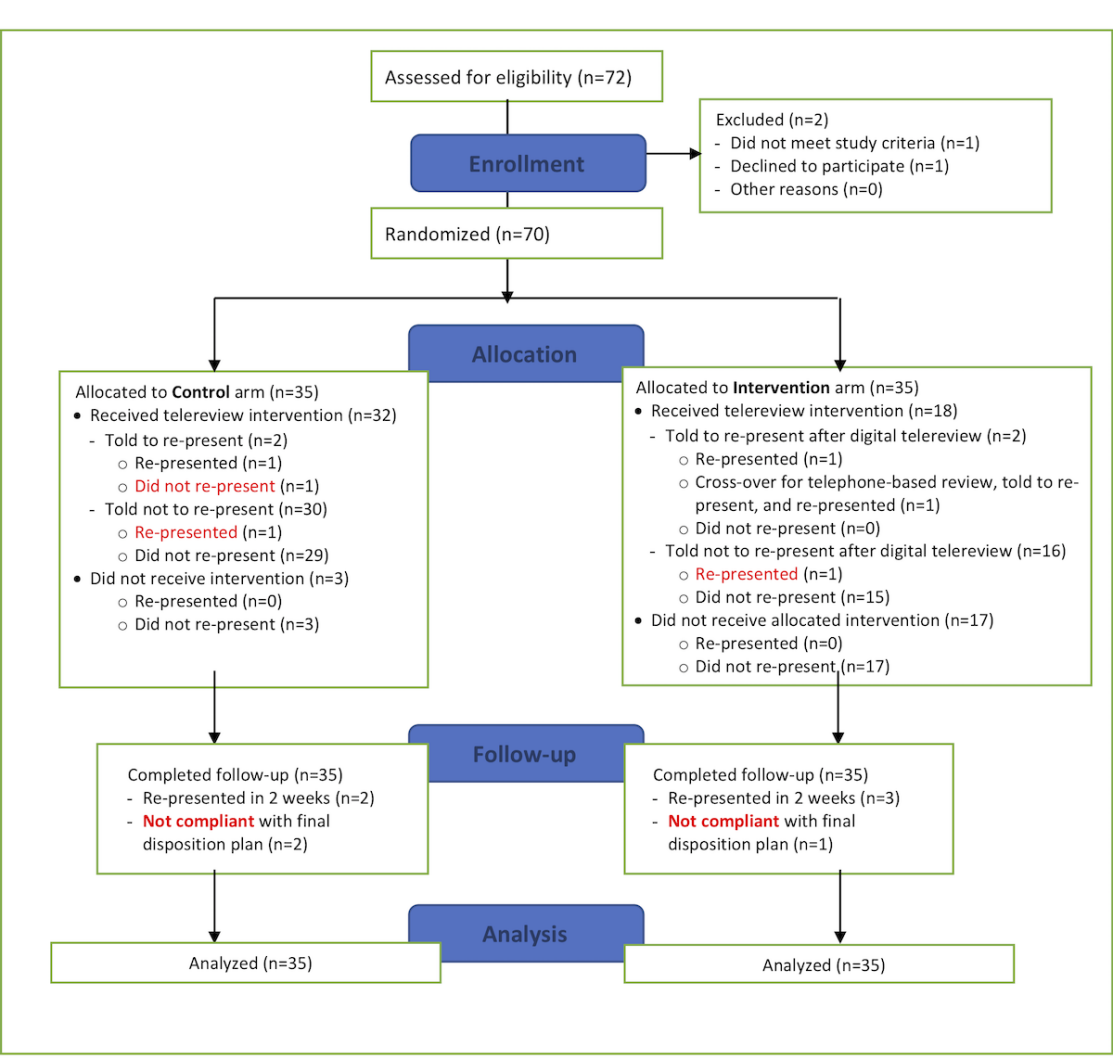
A CONSORT diagram depicting the study. Patients in the control arm receive the push-to-patient form of telephone telereview while patients in the intervention arm receive the pull-from-patient form of digital telereview.

From September 2017 to May 2018, patients (N=72) who were discharged from the emergency department observation ward and who satisfied inclusion criteria were recruited to participate in this study. One patient was under the age of 21 and was excluded. Another patient declined to participate in the study. The remaining patients (N=70) were enrolled with patients (n=35) randomized to each arm (CONSORT diagram, Figure 1). One patient crossed over from digital telereview to telephone-based telereview since, rather than booking a digital telereview appointment, the patient called the emergency department within 72 hours to report persistent symptoms and complied with instructions to return to the emergency department the next day (on the fourth day postdischarge). This patient was analyzed in the intervention arm in accordance with intention-to-treat.

There were no significant demographic differences between the study groups. Demographics of the study population are described in detail in Table 1. Patients were a mean of 40.0 (SD 13.8; range 22-71) years of age, mostly female (47/70, 67%) and mostly of Chinese ethnicity (39/70, 56%). Symptoms reported at initial presentation are detailed in Table 2 and those reported at follow-up are detailed in Table 3 demonstrating the usefulness of a structured web survey in gathering symptomatology during telereview.

**Table 1:** Patient Demographics.

Variable		All (N=70)	DoctorBell (n=35)	Telephone (n=35)		Chi-square (*df*)		*P* value
**Age (years)**					N/A^a^		.88^b^	
	mean (SD)	40.0 (13.8)	40.2 (13.3)	39.7 (14.4)				
	range	22-71	26-69	22-71				
**Gender, n (%)**					0 (1)		>.99	
	Male	23 (33)	12 (34)	11 (31)				
	Female	47 (67)	23 (66)	24 (69)				
**Ethnicity, n (%)**					N/A		0.38	
	Chinese	39 (56)	18 (51)	21 (60)				
	Malay	16 (23)	7 (20)	9 (26)				
	Indian	6 (9)	3 (9)	3 (9)				
	Other	9 (13)	7 (20)	2 (6)				
**Nationality, n (%)**					N/A		0.48	
	Singaporean/Permanent resident	61 (87)	29 (83)	32 (91)				
	Other	9 (13)	6 (17)	3 (9)				
**Pre-study survey respondent, n (%)^c^**					N/A		>.99	
	Patient	56 (92)	28 (93)	28 (90)				
	Caregiver	3 (5)	1 (3)	2 (6)				
	Both	2 (3)	1 (3)	1 (3)				
**Disease manager, n (%)^c^**					N/A		.35	
	Patient	56 (92)	29 (97)	27 (87)				
	Caregiver	0 (0)	0 (0)	0 (0)				
	Both	5 (8)	1 (3)	4 (13)				
**Patient highest level of education, n (%)^c^**					N/A		.38	
	None	1 (2)	0 (0)	1 (3)				
	Primary	2 (3)	2 (7)	0 (0)				
	Secondary	8 (13)	2 (7)	6 (19)				
	Postsecondary Diploma/Certificate	19 (31)	11 (37)	8 (26)				
	Degree	24 (39)	12 (40)	12 (39)				
	Masters/PhD	7 (11)	3 (10)	4 (13)				
**Patient needed relative to accompany to hospital, n (%)^c^**					0.8 (1)		.37	
	Yes	37 (61)	16 (53)	21 (68)				
	No	24 (39)	14 (47)	10 (32)				

^a^N/A: not applicable as Fisher’s exact test was used.

^b^A two-sided *t* test was used here.

^c^This number is less than the group number because respondents did not submit their surveys (n=9, n=5, and n=4 missing in All, DoctorBell, and Telephone, respectively).

**Table 2:** Patient symptoms at presentation to hospital reported in a web-based survey via a link provided following patient recruitment.

Variable	All (n=61)^a^	DoctorBell (n=30)^a^	Telephone (n=31)^a^	Chi-square (*df*)	*P* value
Previous abdominal surgery	14 (23)	5 (17)	9 (29)	0.7 (1)	.40
Abdominal bloating	39 (64)	20 (67)	19 (61)	0 (1)	.87
Loss of appetite	50 (82)	23 (77)	27 (87)	0.5 (1)	.47
Fever	16 (26)	9 (30)	7 (23)	0.1 (1)	.71
Giddiness	34 (56)	16 (53)	18 (58)	0 (1)	.91
Blood in stools	2 (3)	1 (3)	1 (3)	N/A^b^	>.99
Malena	1 (2)	1 (3)	0 (0)	N/A	.49
Diarrhea	35 (57)	16 (53)	19 (61)	0.1 (1)	.71
Pale stools	7 (11)	4 (13)	3 (10)	N/A	.71
Nausea/Vomiting	42 (69)	24 (80)	18 (58)	2.5 (1)	.12

^a^This number is less than the group number because respondents did not submit their surveys (n=9, n=5, and n=4 missing in All, DoctorBell, and Telephone, respectively).

^b^N/A: not applicable as Fisher’s exact test was used.

**Table 3:** Patient symptoms on follow-up teleconsultation reported in a web-based survey via a link provided following patient recruitment.

Variable	All (n=43)^a^	DoctorBell (n=14)^a^	Telephone (n=29)^a^	Chi-square (*df*)	*P* value
Abdominal pain	12 (28)	3 (21)	9 (31)	N/A^b^	.72
Abdominal bloating	8 (19)	3 (21)	5 (17)	N/A	>.99
Diarrhea	12 (28)	3 (21)	9 (31)	N/A	.72
Nausea/vomiting	3 (7)	1 (7)	2 (7)	N/A	>.99
Giddiness	1 (2)	0 (0)	1 (3)	N/A	>.99

^a^This number is less than the group number because respondents did not submit their surveys (n=9, n=5, and n=4 missing in All, DoctorBell, and Telephone, respectively).

^b^N/A: not applicable as Fisher’s exact test was used.

There was an overall 71% (50/70) utilization of the telereview service by 32 patients in the telephone-based telereview control arm (32/35, 91%) and 18 patients in the digital telereview intervention arm (18/35, 51%). Most patients in control (33/35, 94%; 95% CI 79.5%-99.0%) and intervention (34/35, 97%; 95% CI 83.4%-99.9%) arms were compliant with final disposition. There was a low rate of re-presentation at 72 hours for both arms (control: 2/35, 6%; 95% CI 1.0%-20.5%; intervention: 2/35, 6%; 95% CI 1.0%-20.5%) and at 2 weeks for both control (2/35, 6%; 95% CI 1.0%-20.5%) and intervention (3/35, 9%; 95% CI 2.2%-24.2%) arms. After the initial 72-hour period, no patients represented within the control arm crossed over whereas the single patient from the intervention arm who crossed over to telephone-based review re-presented at 4 days. Using the chi-square test, there were no significant differences between the control and intervention arms with regards to efficacy as well as safety in terms of re-presentation within 72 hours and within 2 weeks (*P*>.99).

## Discussion

### Principal Findings

Emergency department overcrowding is a persistent challenge despite increased human resource capacity and process innovations [16] including emergency department observation wards themselves [15]. This has been exacerbated by the COVID-19 pandemic with front-loaded emergency services. New technologies may help resolve overcrowding through automated solutions such as machine learning to optimize existing processes [17] or digital telemedicine to enable new processes that streamline the flow of patients [3]. This study is the first pragmatic randomized controlled trial that evaluated efficacy of digital telereview for the follow-up of patients with undifferentiated acute abdominal pain. This solution uses a hybrid of asynchronous and synchronous teleconsultation—the unique benefits of which have been outlined in a review and case study of their application in eye care [18]. This study is a timely contribution to ongoing debate surrounding the effective implementation of remote consultation in tertiary care. The results of this study suggest that digital telereview may be a safe and effective tool to optimize follow-up processes for right-siting patients in tertiary care.

Digital platforms may enable safe re-design of existing processes from a push-to-patient form of service delivery to a pull-from-patient form. Earlier studies have indicated that patients may not always have adequate insight about the severity of their illness [19]. Hildebrandt et al [19] surveyed primary care physicians regarding patient-directed afterhours telephone triage services. Calls from patients who considered their conditions to be non-emergent were reviewed by physicians who found that roughly half of these patients (range 22%-77%) required urgent review [19]. In this study, telereview following initial physician contact was selected instead of teletriage in order to ensure that all patients were first counselled by a health professional regarding their condition before intervention.

The use of telereview may not be appropriate in the presence of certain clinical factors. These include patient factors such as lack of insight regarding their health or lack of familiarity with the use of mobile phone technology [7,20]. Some researchers posit that dedicated staff should be rostered to respond to any urgent enquiries and actively follow-up all patients to ensure timely assessment [19]; however, the results from this study indicate that patient-led digital telereview following routine education and discharge advice can be as effective as provider-led active follow-up despite the finding of reduced resource utilization (patient-led digital telereview: 18/35, 51%; provider-led telephone-based telereview: 32/35, 91%; Figure 1).

This pull-from-patient service delivery reduces manpower utilization to coordinate unnecessary telereview in patients who are well. Findings from a retrospective study of 1522 video-conference teletriage interventions over 52 weeks support this notion that teleconsultation can improve clinical efficiency [21]. Similarly, Brennan et al [22] reported good outcomes and time saved from emergency department teleconsultations. In this study, the digital platform served as a filter to triage patients, saved time for emergency department staff by collecting fundamental patient-reported history asynchronously, and presented the information accessibly to emergency department staff for clarification with the attending physician at any suitable time before synchronous consultation (instead of traditionally having to place the patient on-hold and interrupt the attending for advice). The results from the current study will facilitate the necessary health economic assessments to guide the implementation of digital telereview, which need to be conducted based on relevant outcomes that have been identified in the literature such as reduced adverse events and reduced time requirement [23].

### Limitations

Limitations of this study include a lack of pre-existing data for sample size calculation and possible selection bias since patients were only recruited when study team members were on shift—a common constraint of studies that are conducted in busy emergency departments [13]. Furthermore, these results may not be generalizable given that participants were relatively young and well-educated although, from a practical perspective, such patients are more likely to use digital consultation services [7,23]. Other limitations are consistent with intention-to-treat analysis such as conservative estimates for efficacy as a result of totals diluted by patients who did not receive the intervention (Figure 1). The strengths of intention-to-treat analysis are that it evaluates the actual performance compared objectively with existing practice by considering any deviations in protocol. Further strengths of this study are the use of the gold standard randomized controlled methodology and the comprehensive description of interventions, participants, and outcomes.

Existing reports caution that successful implementation of a telemedicine intervention may not necessarily be reproduced once the clinical application is even slightly modified. This has been observed with investigations of the WelTel telehealth intervention [2]. Lester et al [2] first reported successful implementation of WelTel for messaging that provides weekly automated notifications for patients with *Human immunodeficiency virus* (and promotes adherence to anti-retroviral therapy with messages based on patient-reported adherence. When van der Kop et al [24] evaluated use of WelTel to promote attendance to follow-up appointments, they found lower compliance in the patients who received the intervention and no significant difference between the groups. Therefore, the results from our study may not be generalizable to other undifferentiated acute presentations such as chest pain. Physicians, administrators, and researchers should be mindful of factors such as clinical context and the form of intervention in the consideration of existing evidence to guide telehealth implementation [20].

The benefits of applying design-thinking in this manner have been described for numerous telehealth applications including tele-ophthalmology enabled by artificial intelligence since contextual considerations such as lack of adequate infrastructure or stable internet access may have an impact on model implementation [25]. In the context of the COVID-19 pandemic, there is a heightened need for providers to evaluate models such as these to rapidly scale health system capacity to address clinical need and to decentralize services to reduce health care–associated transmission [26]. The concurrent use of asynchronous modalities as a filter before synchronous consultation may help ensure better allocation of healthcare manpower similar to the model in this study. Looking beyond the pandemic, when structured appropriately to address the clinical need, digital telereview developed using a design that is mindful of clinical context to guide telehealth implementation (as described above) may lead to considerable cost savings (as suggested by our results). This has already been definitively demonstrated at scale for other related technology applications such as synchronous artificial intelligence–enabled tele-ophthalmology [27] and asynchronous multicenter cloud-computing enabled registry-based research [28].

### Conclusions

Digital telereview may safely facilitate a re-design from a push-to-patient to a pull-from-patient form of follow-up in select patients. This study suggests that there is potential to save cost and manpower-time with digital telereview and has laid the ground work for future investigations to examine the benefits of implementing digital telereview at scale for various clinical applications.

## Appendix

Multimedia Appendix 1 Singapore General Hospital (SGH) Emergency Observation Ward Gastroenteritis and Abdominal Pain protocols.

Multimedia Appendix 2 CONSORT-eHEALTH checklist (V 1.6.1).

## Abbreviations

COVID-19: coronavirus disease 2019
SAVED: Safety and efficacy of follow-up for patients with abdominal pain using video consultation
