# Supplementary material for: Evaluating Safety and Efficacy of Follow-up for Patients With Abdominal Pain Using Video Consultation (SAVED Study): Randomized Controlled Trial
Source: J Med Internet Res. 2020 Jun 15;22(6):e17417. doi: 10.2196/17417 (PMC7324993; doi:10.2196/17417)
Supplement: Multimedia Appendix 1 [file jmir_v22i6e17417_app1.pdf]

Here we append the inclusion criteria for the Singapore General Hospital (SGH) Emergency Observation Ward for patients under the Gastroenteritis and Abdominal Pain protocols who were considered for participation in this study:

1) Abdominal pain protocol (page 2)
2) Gastroenteritis protocol (page 3-4)

Thank you for your kind consideration.

EOW: ABDOMINAL PAIN

ADMITTING DEM DOCTOR

Inclusion Criteria

- ◻ Abdomen soft, no guarding/ rebound, bowel sounds present
- ◻ Haemodynamically stable

Exclusion Criteria

- ◻ Haemodynamically unstable: HR >120 and/or SBP <90
- ◻ Signs of acute abdomen/surgical cause e.g. guarding, rebound, pulsatile mass
- ◻ Suggestion of ischaemic bowel, especially in high risk patients (consult ED senior)
  - o Elderly patients with IHD, AF, HTN, DM, HLD
- ◻ Suggestion of ectopic pregnancy and UPT positive
- ◻ Signs of BGIT e.g. PR bleeding/ malaena, hematemesis
- ◻ Non-abdominal causes of abdominal pain e.g. pneumonia, DKA
- ◻ Markedly deranged blood investigations
- ◻ Paediatric patients and patients from community hospital

ED Investigations

- ◻ POCT: ECG, UC9, UPT, H/C
- ◻ Bloods: FBC, UECr, LFT, Amylase
- ◻ Imaging: CXR (erect) ± AXR (supine) / KUB

EOW DOCTOR AND RESIDENT NURSE

EOW Intervention

- ◻ Vital signs Q2H, clinical review Q3H
- ◻ Input/output chart if IV fluids given
- ◻ NBM as necessary
- ◻ Consider advanced imaging – refer to DEM Handbook for workflow regarding CTAP
- ◻ Medications
  - o Anti-spasmodic e.g. Buscopan
  - o Anti-emetic e.g. Maxolon or Stemetil
  - o Oral medications e.g. MMT or Charcoal
  - o PPI e.g. IV Omeprazole

Discharge Criteria

- ◻ Resolution of abdominal pain
- ◻ Able to tolerate meals/feeds
- ◻ Haemodynamically stable

Admission Criteria

- ◻ Persistent abdominal pain with guarding or rebound

Paste patient's label here

EOW: GASTROENTERITIS

ADMITTING DEM DOCTOR

Inclusion Criteria

- ☐ Gastroenteritis: Diarrhoea and/or vomiting and/or abdominal colic
- ☐ Clinical signs of dehydration
  - o Postural hypotension
  - o Decreased skin turgor
  - o Tongue dry, sunken orbits, dry mucosal membranes

Exclusion Criteria

- ☐ Abnormal vital signs
  - o SpO2 <95% and/or Respiratory rate >25/min
  - o SBP <80mmHg and/or Pulse >110/min
  - o Temp < 36 or > 39
- ☐ Toxic/ Septicaemic
- ☐ Bloody or infectious diarrhoea
- ☐ Complicated diabetes mellitus: DKA, HHNK, hypoglycaemia
- ☐ Abdominal pathology e.g. intestinal obstruction, ischaemic bowel, bleeding GIT
- ☐ Comorbidities: CCF, CVA, neoplastic disease, renal disease, liver disease (soft contraindication)
- ☐ POCT findings
  - o Abnormal UC9 (UTI, ketones+)
  - o Positive UPT
  - o Abnormal ECG
- ☐ Laboratory findings
  - o FBC: TW > 20K, HCT >50, Plt <100K
  - o UECr: Urea >7.7mmol/L, Sodium <130mmol/L, Bicarbonate <20mmol/L
- ☐ Paediatric patients

ED Investigations

- ☐ POCT: ECG, CBG, UC9, UPT (in females)
- ☐ Bloods: FBC, UECr

EOW DOCTOR AND NURSES

EOW Interventions

(Please tick the boxes after they have been done)

- ☐ Vital signs Q2H
- ☐ Clinical review Q3H
- ☐ IV rehydration with Normal Saline 500-2000mls over 8-23hrs (depending on degree of dehydration, caution in elderly with co-morbidities)
- ☐ Document Intake and output Q2H
- ☐ Oral Rehydration Salts as well as oral fluids
- ☐ Administer medications e.g. Antipyretics, Charcoal, Maxalon, Buscopan as necessary

Discharge Criteria

- ☐ Vital signs stable
- ☐ Cessation of symptoms
- ☐ Able to tolerate food and drinks

If for discharge

- ☐ Symptomatic medications where necessary e.g. Charcoal 2 tabs TDS, Buscopan 10mg TDS, ORS 1 sachet TDS, Maxolon 10mg or Stemetil 12.5mg TDS
- ☐ Dietary advice (avoid milk products till diarrhoea settles, encourage soft diet, ORS, clear fluid)
- ☐ Medical leave
- ☐ Refer OPS for f/up if >40yrs, hx of DM
- ☐ Return advice if patient has recurrent symptoms, increasing abdo pain, persistent vomiting or bloody stool

Admission Criteria

- ☐ Admit to Acute Medical Ward if
  - Persistent symptoms
  - Persistent postural BP drop after intravenous hydration
  - Unable to tolerate feeds
- ☐ Admit to GS if
  - Worsening of abdominal pain during review periods
  - Signs suggestive of acute abdomen

Updated Dec 2017
